# Cardiorespiratory and metabolic responses and reference equation validation to predict peak oxygen uptake for the incremental shuttle waking test in adolescent boys

**DOI:** 10.1371/journal.pone.0206867

**Published:** 2018-11-01

**Authors:** Andreza L. Gomes, Vanessa A. Mendonça, Tatiane dos Santos Silva, Crislaine K. V. Pires, Liliana P. Lima, Alcilene M. Silva, Ana Cristina R. Camargos, Camila D. C. Neves, Ana C. R. Lacerda, Hércules R. Leite

**Affiliations:** 1 Programa de Pós-Graduação em Reabilitação e Desempenho Funcional, Departamento de Fisioterapia, Universidade Federal dos Vales do Jequitinhonha e Mucuri (UFVJM), Campus JK, Alto da Jacuba, Diamantina, Minas Gerais, Brazil; 2 Escola de Educação Física, Fisioterapia e Terapia Ocupacional (EEFFTO), Departamento de Fisioterapia, Universidade Federal de Minas Gerais (UFMG), Diamantina, Minas Gerais, Brazil; UFMG, BRAZIL

## Abstract

**Background:**

Previous studies speculated that the Incremental Shuttle Walking Test (ISWT) is a maximal test in children and adolescents, however comparison between ISWT with cardiopulmonary exercise test has not yet performed. Furthermore, there is no regression equation available in the current literature to predict oxygen peak consumption (VO_2_ peak) in this population. This study aimed to assesses and correlate the cardiorespiratory responses of the ISWT with the cardiopulmonary exercise (CEPT) and to develop and validate a regression equation to predict VO_2_ peak in healthy sedentary adolescent boys.

**Methods:**

Forty-one participants were included in the study. In the first stage, the VO_2_ peak, respiratory exchange ratio (R peak), heart rate max (HR max) and percentage of predicted HR max (% predicted HR max) were evaluated in CEPT and ISWT (n = 26). Second, an equation was developed (n = 29) to predict VO_2_ peak. In both phases, the VO_2_ peak, respiratory exchange ratio R and hearth rate (HR) were evaluated. In the third stage, the validation equation was performed by another 12 participants.

**Results:**

Similar results in VO_2_ peak (P>0.05), R peak (P>0.05) and predicted maximum HR (P>0.05) were obtained between the ISWT and CEPT. Both tests showed moderate significant correlations of VO_2_ peak (r = 0.44, P = 0.002) e R peak (r = -0.53, P < 0.01), as well as the agreement of these measurements by Bland-Altman analysis (VO_2_ peak, bias = -0.13; R peak, bias = 0.0). Distance walked was the variable that explained 42.5% (R^2^ = 0.425, p = 0.0001) of the variance in VO_2_ peak. The equation was VO_2_ peak (predicted) = 20.94 + (0.02 x distance walked). The results obtained by the equation were not significantly different compared to the values obtained by the gas analyzer and the Bland-Altman analysis showed agreement (bias = 1.6).

**Conclusion:**

The ISWT produced maximal cardiorespiratory responses comparable to the CEPT, and the developed equation showed viability for the prediction of VO_2_ peak in healthy sedentary adolescent boys.

## Introduction

Assessment of functional capacity or cardiorespiratory fitness (CRF) is defined as the ability to perform a moderate to high intensity exercise involving large muscle groups over a period of time [1,2]. It is an important component of health related physical fitness, which reflects the functional capacities of the respiratory, cardiovascular and musculoskeletal systems [1]. The CRF assessment has been widely used in clinical practice and research aiming to provide parameters for physical activity prescription and to evaluate reduced exercise tolerance in several health conditions [3–5].

The performance of a cardiopulmonary exercise testing (CEPT) followed by the measurement of peak oxygen consumption (VO_2_ peak) through the direct analysis of exhaled gases is the most commonly reported procedure in the literature for the evaluation of CRF [6]. However, this measurement is often infeasible because its require high-cost equipment, specialized laboratory and trained professionals [7]. Thus, field test and prediction equation to indirect estimate VO_2_ peak in clinical practice has been widely implemented [2]. Among the field test, we highlight the Incremental Shuttle Walking Test (ISWT) developed by Sing et al., [8] which comprises as a simple incremental walk test with pace dictated by external stimulus which asses CRF based on distance walked. Despite being developed initially for individuals with chronic obstructive pulmonary disease [8], it has been used recently in different health conditions and age groups [5,9,10].

Some studies have used ISWT to asses CRF in children and adolescents with asthma [11], scoliosis [5] and very low premature newborn [10]. However, the application and intensity of this test in a healthy population is scarce. Lanza et al., [9] developed an equation to predict distance walked and also showed that the ISWT is a maximal test in a children and adolescent population determined indirectly by means of the maximum heart rate (HR max) achieved at the end of the test. On the other hand, Coelho et al. [12] demonstrated that a healthy control group of children and adolescents showed submaximal values of HR max in the ISWT. However, these authors fail to confirm the cardiorespiratory responses with the completion and comparison with the CEPT.

Taken together, there is a gap in the current literature regarding the ISWT intensity validation, as well as an equation to predict VO_2_ peak in the adolescent population. Thus, the present study aims to evaluate and correlate the cardiorespiratory outcomes during the ISWT and a CEPT, in order to classify the intensity of ISWT, and to develop and validate an equation to predict VO_2_ peak in healthy adolescent boys. We postulate that the ISWT would promote maximal cardiorespiratory responses in agreement with the CEPT, and the regression equation would be feasible for predicting VO_2_ peak in healthy adolescent boys.

## Materials and methods

This was a cross-sectional study that included 41 healthy adolescent boys. They were recruited by convenience from private and public schools. The protocol began in July 2016 and ended in November 2017. All measurements were obtained in the physiology of exercise laboratory (UFVJM) by trained investigators. The parents were asked to report the health history of the subject (i.e., prematurity birth and current physical activity engagement and comorbidities). Thus, the inclusion criteria were as follows: male boys, ages 12–18 years old, absence of chronic or acute diseases, physical activity engagement less than three times a week, preterm birth and parents sign the consent form. The volunteers were excluded if they were unable to understand the test. To meet the objectives, this study was divided into three stages. The first stage aimed to evaluate the intensity of the ISWT; the second stage aimed to develop a regression for the prediction of VO_2_ peak; and the third stage to validate the prediction equation. This study followed the declaration of Helsinki. The Ethics and Research Committee of Universidade Federal dos Vales do Jequitinhonha e Mucuri (UFVJM), Brazil, approved this study (Protocol: 52980816.4.0000.5108). The following protocol description reproduces information already reported elsewhere [2].

### First stage procedures

In the first stage, 26 volunteers went to the laboratory on three consecutive days at the same time period each day. On the first day, the body composition was assessed (weight, height, BMI) and familiarization was performed. The weight and height were measured on an anthropometric mechanical scale, the BMI was calculated as the weight divided by height squared [3]. Familiarization consisted of testing that would be performed on consecutive days to reduce the effect of learning. On the second and third days, the ISWT and CEPT were applied. The testing order was randomized and balanced. The subjects were instructed to avoid physical activity and any intake of caffeine in the 24 hours prior to testing, to get at least 8 hours of sleep the night before, to eat a light meal and to ingest 500 ml of water in two hours before the tests. On the days of testing subjects were asked about their compliance with the recommendations above and about possible complications or changes in their daily routines [2].

To perform the ISWT, the participants were instructed to walk a distance of 10 meters around a marking between two cones, placed 0.5m from each endpoint [8]. The walking speed at which the participant should walk (or run) was dictated by a sound played from a CD that was originally generated by a microcomputer. Each minute the walking speed increased by 0.17m/s. The test was finished when the volunteer was not able to maintain the required speed (more than 0.5m from the cone), at the request of the volunteer, or for some other reported symptom (dyspnea, dizziness, vertigo, angina). The original protocol consisted of 12 levels (1020m); however, as suggested by the literature, we used a protocol of 15 levels (1500m) to evaluate healthy participants, in order to prevent the ceiling effect [13]. Additionally, during the testing, the laps were recorded to calculate the distance and gait speed reached at the last full level. The ISWT were performed twice with at least 30 min of rest between them. The best test (i.e., the longest distance walked) was considered for analysis. The maximum difference between the tests should be 40 m [14]. A third test was performed when the difference was greater than this. A trained professional conducted the tests. Before and after the test, heart rate (HR, measured by a heart rate monitor) and blood pressure (measured by a mercury sphygmomanometer cuff and a stethoscope) were measured.

The CEPT was performed on a treadmill using a protocol based on the progression of the ISWT. This protocol consisted of 1-minute stages, with speed increasing every minute without increasing the incline of the treadmill. The initial speed was 0.5 m/s, and it increased by 0.17 m/s at each stage. Before, during and after the test, heart rate and blood pressure were measured as described above. The criteria for stopping the test was as follows systolic blood pressure (SBP) greater than 210 mmHg; diastolic blood pressure greater than 120 mmHg; sustained decrease in SBP, angina dyspnea, cyanosis; nausea, dizziness or by the request of the volunteer [15].

### Second stage procedures

In the second stage 29 volunteers went to the laboratory at two different days. On the first day, the body composition measurements were obtained as described in the first stage. On the second day, the participants went to the laboratory for two ISWT with an interval of 30 minutes between them. Completion of two ISWTs with this interval had been suggested to reduce the effects of the learning test [13,16]. For the data analysis, the results of the test in which the volunteer obtained the greatest distance covered were used. As with the first stage, the entire procedure took place during a single day shift: the subjects were instructed to follow all the recommendations for the practice of physical tests, and prior to completion of the tests.

### Cardiorespiratory and metabolic responses

During the tests of the two stages of this study, the exhaled gases were collected using a gas analyzer via the portable telemetry system (k4b2, Cosmed, Rome, Italy). Among other variables, oxygen uptake (VO_2_), respiratory quotient (R) and HR breath-by-breath were monitored. The absolute VO_2_ peak rate (mL/min) was expressed as relative rate defined as VO_2_ peak (mL/kg/min) and R peak the highest value of these measures at peak effort [17] and maximum heart rate (HRmax) as the highest HR value recorded during the test [2]. The maximum predicted HR was calculated as 208 (0.7 * age) [18].

### Validation of the reference equation

To validate the equation, a different group of healthy males, composed of 12 individuals, was selected according to the same inclusion criteria of the study. This group completed the ISWT as described in the preceding stages. Likewise, the VO2 peak was predicted by the reference equation.

### Statistical analysis

The statistical analysis was performed using the statistical packages SPSS 22.0 (Inc., USA) and GraphPad Prism 4 (Inc., USA). In the first stage, the normality of data was checked by the Shapiro–Wilk test and the differences among measured variables were determined by paired-t-test for variables with normal distribution or the Wilcoxon test for variables with non-normal distribution. Pearson’s coefficient of correlation was performed to study the correlation between variables and the agreement between tests was assessed by Bland-Altman analysis. The sample size was calculated based on the study by Neves et al [2] and was identified at least 10 participants. In the second stage, the normality of data was checked by Kolmogorov-Smirnov test and for compiling the reference equation, the linear multiple regression analysis was performed to identify the predictors of the dependent variable. Multicollinearity was measured by variance inflation factors (VIF). In this stage, the sample size was estimated on GPower Software version 3.1 and based on the relationship between the numbers of variables to be included in the multiple regression analysis and the minimum number of observations required, indicating at least 29 participants in order to develop a linear model containing up to three variables. At the end of the regression analysis, the paired t-test was utilized to compare the means of the results obtained by the reference equation with the measured values of VO2 peak obtained using the gas analyzer. Moreover, the validation of the reference equation was evaluated in an additional group of 12 volunteers: the values of VO2 peak obtained by the reference equation were compared with the measured values of VO2 peak obtained by the gas analyzer using the paired t-test. The level of statistical significance was P<0.05.

## Results

A total of 336 subjects were screened, but 186 did not return the baseline questionnaire. From the 150 eligible participants, 28 reported any chronic, acute illness or reported premature birth, 49 decline and 32 subjects were excluded for other reasons. The final sample was 41 male adolescents.

### First stage

**Fig 1:**
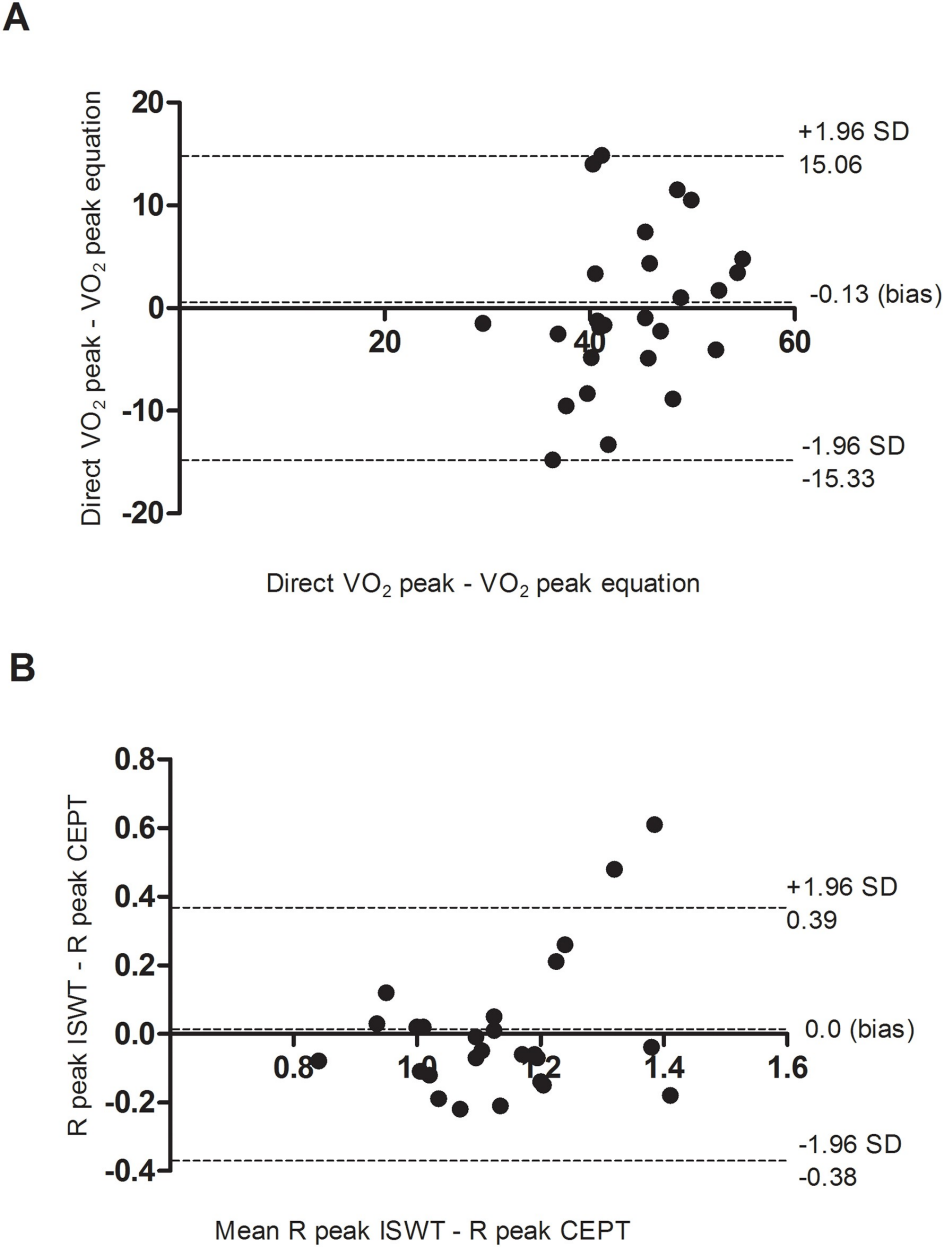
Agreement between VO_2_ (mL/kg/min) peak and R peak obtained in the ISWT and CEPT. (A) Bland-Altman plot of the difference between the VO_2_ peak of the ISWT and CEPT plotted against the mean VO_2_ peak of the ISWT and CEPT; (B) Difference R peak of the ISWT and CEPT plotted against the mean R peak of the ISWT and CEPT. ISWT = Incremental Shuttle Walking Test; CEPT = cardiopulmonary exercise testing; VO_2_ = oxygen uptake; R = respiratory exchange ratio.

The general characteristics of the participants of first and second stage and their performance on ISWT are showed in Table 1. The cardiorespiratory responses obtained at the end of the ISWT and CEPT are presented in Table 2. Similar results in VO_2_ peak, R peak, and predicted HRmax were found. Moderate and significant correlations in VO_2_ peak (r = 0.44, P = 0.02) and R peak (r = -0.53, P<0.01) were found between the tests. The Bland-Altman analysis also showed agreement between the results for VO_2_ peak (bias = -0.13) and R peak (bias = 0.00) on the ISWT and CEPT (Fig 1A and 1B).

**Table 1:** Characteristics of participants of the first, second and third stage.

Characteristics of the participants (n = 41)	First phase (n = 26)	Second phase (n = 29)	Third phase (n = 12)
Age *(yr)*	14.2 (1.8)	14.3 (1.8)	14.3 (1.9)
BMI *(kg/m*^*2*^*)*	19.5 (2.4)	19.5 (2.4)	20.5 (2.8)
Gait speed (m/s)	2.2 (0.3)	2.2 (0.3)	2.2 (0.4)
Distance walked (m)	923.9 (249.4)	938.0 (250.2)	915 (309.5)

The data is presented as mean (SD). BMI = body mass index.

**Table 2:** Comparison between the results of cardiorespiratory variables at the end of the test, obtained in ISWT and CEPT.

Outcome	Tests		Comparison between tests
	ISWT (n = 26)	CEPT (n = 26)	*P*-value
VO_2_ peak (mL/kg/min)	44.02 (8.2)	44.2 (6.2)	0.93^a^
R peak	1.1 (0.2)	1.1 (0.1)	0.28^b^
HR max (% predicted)	98.8 (6.3)	96.6 (3.5)	0.63^b^

The data is presented as mean (SD). ISWT = incremental shuttle walking test; CEPT = cardiopulmonary exercise testing; VO_2_ = oxygen uptake; R = respiratory exchange ratio; HR = heart rate.

^a^Paired-*t* test,

^b^Wilcoxon test.

### Second stage

The characteristics of the participants of the second stage are showed in Table 1. Considering the best ISWT, age, BMI and distance walked were the demographic, anthropometric and physical performance variables selected for the preparation of the reference equation, respectively. The univariate analysis showed that the VO_2_ peak correlated significantly with age (r = 0.38, p = 0.04), and distance (r = 0.67, p = 0.0001). There was no significant correlation with BMI (r = -0.24, p = 0.22). A model of stepwise linear multiple regressions showed that distance walked explained 42.5% (R^2^ adjusted = 0.425, p = 0.0001) of the variance in VO_2_ peak. The 95% Confidence Interval for unstandardized coefficients were the constants (11.12 to 30.77) and distance (0.01 to 0.03). The reference equation for the VO_2_ peak in the ISWT was:

$${\text{VO}}_{2}\;\text{peak}(\text{predicted})=20.94+(0.02\;\text{x}\;\text{distance}\;\text{walked})$$

### Validation of the reference equation

**Fig 2:**
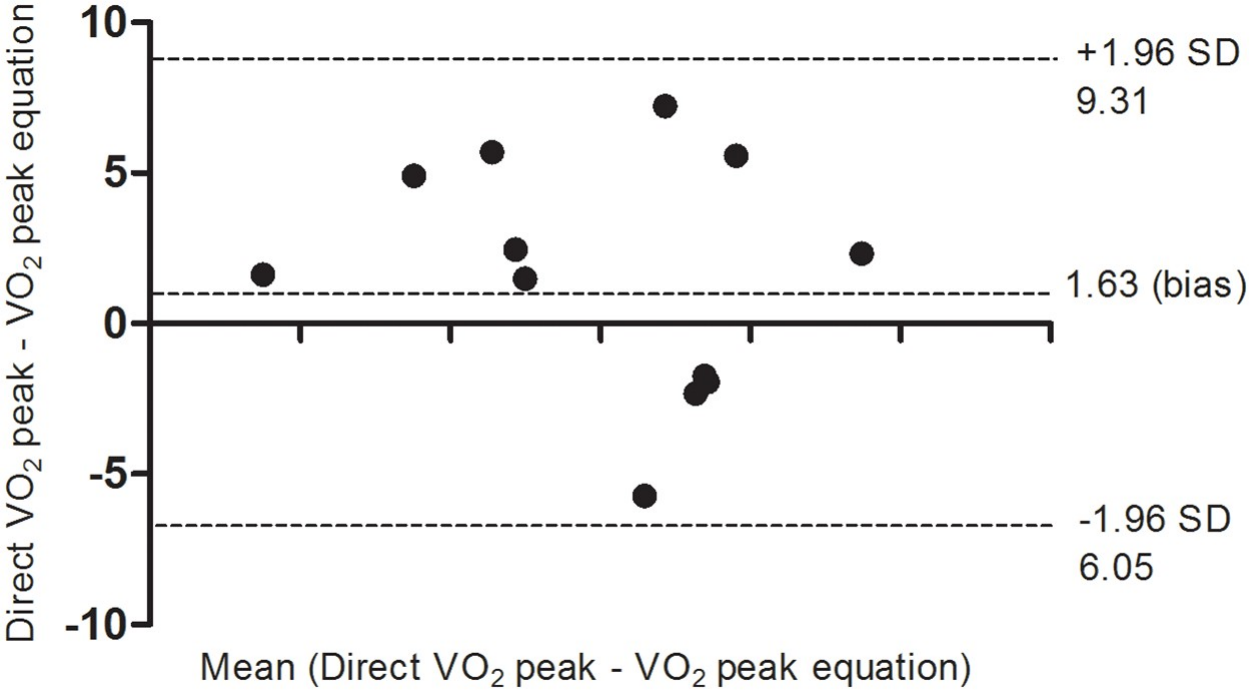
Bland-Altman agreement of VO_2_ peak in the validation of the reference equation.

The characteristics of the volunteers who attended in the equation validation stage were present in Table 1. The results obtained by the equation of VO_2_ peak with the values obtained by the gas analyzer, showed no significant difference between them (VO_2_ peak [predicted] = 39.24 ± 6.1 mL/kg/min; VO_2_ peak [gas analyzer] = 40.87 ± 5.4 mL/kg/min, P = 0.1776). It was possible to verify the agreement between these measures by the Bland-Altman method, in which a bias of 1.6 was showed, representing a difference of 4.4% in the VO_2_ peak (Fig 2). Furthermore, there was no statistically significant difference between the participants of equation elaboration and validation for age (p = 0.7978), weight (p = 0.5498), height (p = 0.0650), BMI (p = 0.2480), distance walked (p = 0.9213) and walking speed (p = 0.0.6212).

## Discussion

The present study describes the comparison of CRF between the ISWT with CEPT in healthy sedentary adolescent boys. In the ISWT, the adolescent boys reached values of HRmax > 90% and R peak > 1.1, thus classifying the ISWT as a maximal effort test [19,20]. Furthermore, results showed a moderate and significant correlations as well as agreement between VO_2_ peak and R peak by both tests. Our results are corroborated by the results of Lanza et al. [9]. These authors showed that ISWT is a maximal test in children and adolescent by registering higher HR values (>90%) at end of the test. Our research group also showed previously in healthy men [2] and women (data not published) that cardiorespiratory outcomes (VO_2_ peak and R peak) collected during ISWT are comparable to CEPT test, as well as both tests showed agreement and high correlations between VO_2_ and R peak between ISTW and CEPT [2]. In the other hand, previous studies showed lower HR max in healthy control children at end of the test compared to our data, such as 69% [11] and 55% [10]. However, it’s important to highlight that our participants were allowed to run which can explain the higher HRmax found. To the best of our knowledge there are no studies evaluating CRF between ISWT and CEPT in healthy sedentary adolescent boys. Taken together, our data support that ISWT can be considered as a valid measure to assess CRF in this population as a maximal effort test.

Additionally, our study is the first one to develop an equation to predict VO_2_ peak in the ISWT in this population. Despite of have including anthropometric variables in the multivariate analysis, only distance walked explained the variance of VO_2_ (43%) peak in our population. Distance walked as one of the major determinant of VO_2_ peak was also observed in previous studies that developed reference equations for the prediction of VO_2_ peak during the application of the ISWT in healthy adults [21,22] and during the six-minute walking test in obese adolescents [23].

Although the age was significantly correlated to VO_2_ in the linear analysis, this correlation was not strong the sufficient for explained the variance of VO_2_ peak. Similar result was observed by Tsiaras et al. (2010), which shown that the addiction of age did not further improve the prediction accuracy of the equation for prediction of VO_2_ peak from a maximal treadmill test in 12–18 year-old active male adolescents [24]. This absence of influence of age seems to be related to the stabilization of aerobic performance in youth when compared to childhood. In fact, previous studies showed that the performance of adolescents improved linearly with increase of age, it increased up to 12–13 years, and after (aged 14–19 years) tended to achieve a plateau [25,26]. As with age, BMI did not influence the prediction of VO_2_ peak. The probable reason for this seems to be related to homogeneity of sample of present study. It is noted that participants of present study showed normal BMI. Thus, given that the CRF is lower in adolescents who are overweight than in those of normal weight, the normal body composition did not was correlated to VO_2_ peak [25,27]. Finally, it’s important to highlight that distance walked is a feasible variable in clinical practice and have to take into account when developing a regression equation [28].

Although the prediction equation proposed in the present study might be explained by moderate variance, the VO_2_ peak data collected by the gas analyzer and the developed equation showed agreement. Moreover, the reference values from the current literature that classify CRF (i.e. very week to excellent) vary approximately 7mL/kg/min among the age ranges. Thus, the variation found in the present study (4%) is less likely to change the individuals CRF classification [17]. Finally, the VO_2_ peak mean reached by the male adolescents in our study (~ 44.0 mL/kg/min) was smaller than previous study reporting VO_2_ reference for trained men with age ranging from 15 to 24 (53.3 mL/kg/min) [17] or 10–14 years old (≥ 52.3 mL/kg/min) [6], which classifies our population as sedentary [17].

The results pointed here raise important advancing scientific knowledge regarding the level of ISW in healthy sedentary adolescent boys. The results found in this study contribute to the process of measurement of peak VO_2_ becomes more accessible to clinical practice so that the prescription and elaboration of exercise programs happen in a more informed and assertive way, as well as ISWT can be used as a maximal effort test in replacement of submaximal field test available (e.g. six minute walking test) [29]. Moreover, clinicians should considerer ISWT instead of other field test (e.g. 9-minute walk / run test, 1-mile walk / run test and the 20 m Shuttle Run Test) [30–32] because these tests are influenced by external factors (e.g. motivation and self-paced) which can lead to great variability and compromising the application in randomized controlled trials. Lastly, our prediction equation could be used in clinical studies aiming to investigate CRF in disable adolescent boys population avoiding to use control groups for comparing theirs results [5,9,10]. However, due to restrictions of funding and time, no further experiments such as assessing girls and children with age under 12 years old were conducted. Further studies are necessary to address this population.

## Conclusion

In a conclusive way, the VO_2_ peak values found in our study allow us to affirm that the ISWT was in fact a maximum intensity test in healthy sedentary adolescent boys assessed by direct gas analyzer. Furthermore, the regression equation was feasible and might be useful for clinicians for predicting VO_2_ peak in this population.
