# Correction: Cardiorespiratory and metabolic responses and reference equation validation to predict peak oxygen uptake for the incremental shuttle waking test in adolescent boys

**DOI:** 10.1371/journal.pone.0208826

**Published:** 2018-12-12

**Authors:** Andreza L. Gomes, Vanessa A. Mendonça, Tatiane dos Santos Silva, Crislaine K. V. Pires, Liliana P. Lima, Alcilene M. Gomes, Ana Cristina R. Camargos, Camila D. C. Neves, Ana C. R. Lacerda, Hércules R. Leite

The sixth author’s last name was incorrect. The correct name is Alcilene M. Gomes. The correct citation is: Gomes AL, Mendonça VA, Santos Silva Td, Pires CKV, P. Lima L, Gomes AM, et al. (2018) Cardiorespiratory and metabolic responses and reference equation validation to predict peak oxygen uptake for the incremental shuttle waking test in adolescent boys. PLoS ONE 13(11): e0206867. https://doi.org/10.1371/journal.pone.0206867
